# Promoter Methylation Pattern Controls Corticotropin Releasing Hormone Gene Activity in Human Trophoblasts

**DOI:** 10.1371/journal.pone.0170671

**Published:** 2017-02-02

**Authors:** Xin Pan, Maria Bowman, Rodney J. Scott, John Fitter, Roger Smith, Tamas Zakar

**Affiliations:** 1 Mothers and Babies Research Centre, Hunter Medical Research Institute, New Lambton Heights, NSW, Australia; 2 Priority Research Centre for Reproductive Science, Faculty of Health and Medicine, The University of Newcastle, Callaghan, NSW, Australia; 3 Molecular Medicine, Hunter Area Pathology Service, New Lambton Heights, NSW, Australia; 4 John Hunter Hospital, New Lambton Heights, NSW, Australia; University of Illinois at Urbana-Champaign, UNITED STATES

## Abstract

Placental CRH production increases with advancing pregnancy in women and its course predicts gestational length. We hypothesized that *CRH* gene expression in the placenta is epigenetically controlled setting gestational trajectories characteristic of normal and pathological pregnancies. Here we determined histone modification and DNA methylation levels and DNA methylation patterns at the *CRH* promoter in primary trophoblast cultures by chromatin immunoprecipitation combined with clonal bisulfite sequencing and identified the transcriptionally active epialleles that associate with particular histone modifications and transcription factors during syncytialisation and cAMP-stimulation. *CRH* gene expression increased during syncytial differentiation and cAMP stimulation, which was associated with increased activating and decreased repressive histone modification levels at the promoter. DNA methylation levels remained unchanged. The nine CpGs of the *CRH* proximal promoter were partially and allele-independently methylated displaying many (>100) epialleles. RNA-polymerase-II (Pol-II) bound only to three particular epialleles in cAMP-stimulated cells, while phospho-cAMP response element-binding protein (pCREB) bound to only one epiallele, which was different from those selected by Pol-II. Binding of TATA-binding protein increased during syncytial differentiation preferentially at epialleles compatible with Pol-II and pCREB binding. Histone-3 acetylation was detected only at epialleles targeted by Pol-II and pCREB, while gene activating histone-4 acetylation and histone-3-lysine-4 trimethylation occurred at *CRH* epialleles not associated with Pol-II or pCREB. The suppressive histone-3-lysine-27 trimethyl and–lysine-9 trimethyl modifications showed little or no epiallele preference. The epiallele selectivity of activating histone modifications and transcription factor binding demonstrates the epigenetic and functional diversity of the *CRH* gene in trophoblasts, which is controlled predominantly by the patterns, not the overall extent, of promoter methylation. We propose that conditions impacting on epiallele distribution influence the number of transcriptionally active *CRH* gene copies in the trophoblast cell population determining the gestational trajectory of placental CRH production in normal and pathological pregnancies.

## Introduction

Corticotropin releasing hormone (CRH) concentration increases exponentially in the maternal plasma during the third trimester of pregnancy. Spontaneous preterm birth is associated with an accelerated rise, while post-term pregnancies are characterised by a significantly retarded increase of CRH level in the maternal circulation [1]. The trajectory of the change is predictive of gestational length suggesting that the process controlling maternal CRH levels may be linked to the mechanism that determines the time of birth.

The source of plasma CRH in pregnancy is the syncytial layer of the placenta [2]. It has been proposed that CRH output is accelerated with advancing gestation because of the increasing syncytial content of the placenta and the feed-forward action of glucocorticoids, which upregulates *CRH* gene expression in trophoblasts [3]. Furthermore, there is strong evidence that cAMP is a powerful stimulant of *CRH* gene activity in cultured trophoblast cells supporting the possibility that trophoblast-derived hormonal factors acting via cAMP, such as hCG, may also drive *CRH* expression in an autocrine feed-forward manner [4, 5]. Studies using transfected *CRH* promoter-reporter constructs have indicated that *CRH* gene regulation in trophoblast cells depends on complex molecular interactions centered around a canonical cAMP response element (CRE) in the proximal promoter [6]. A second, placenta-specific cAMP-response element and an NF-kappa-B response sequence have also been implicated in *CRH* expression control under various basal, glucocorticoid- and cAMP-stimulated conditions [7–9].

Recent studies revealed an additional level of *CRH* gene regulation involving epigenetic chromatin modifications in the promoter region. In the central nervous system of rats and mice, *CRH* gene activity is inhibited by DNA methylation and histone deacetylation in the *CRH* promoter region, while in human trophoblasts, dynamic histone-3, lysine-9 acetylation at the *CRH* promoter has been shown to underpin the glucocorticoid stimulation of *CRH* expression [10–12]. A regulatory role of DNA methylation at CpG dinucleotides is supported by the comprehensive analysis of the placental methylome, which has placed the *CRH* gene in a highly methylated domain of the trophoblast genome containing genes with tissue specific expression [13]. Furthermore, the *CRH* proximal promoter is located within a CpG island shore region (relative to the intragenic CpG island), which is typically associated with tissue-specific differential methylation and gene expression [14]. The proximal promoter of the human *CRH* gene contains 9 CpG dinucleotides, which can be methylated. These CpGs are conserved in the mouse, rat and the human including their positions within the promoter sequence [6]. Notably, one of the CpGs in the *CRH* proximal promoter is located within the CRE. Methylation of the CpG within the CRE has been shown to have major effects on the transcription factor binding properties of the sequence, diminishing its affinity to pCREB (phosphorylated cAMP response element binding protein) and enhancing its activity to bind CEBPA (CCAAT/Enhancer binding protein, alpha) with profound functional consequences on gene expression control [15]. Based on this information, we hypothesized that CpG methylation in the promoter regulates *CRH* gene activity in human trophoblasts by influencing transcription factor binding and the deposition of histone modifications that control chromatin structure coordinately with DNA methylation. We used primary human trophoblast cells in the experiments, which differentiate to syncytia in culture and respond to cAMP stimulation with robust increase of *CRH* expression [5]. Combining chromatin imunoprecipitation (ChIP) with clonal bisulfite sequencing allowed us to determine CpG methylation with single base resolution in individual *CRH* promoters in subpopulations of *CRH* gene copies bound to transcription factors or marked with various histone modifications. The results uncovered the epigenetic and functional heterogeneity of the *CRH* promoter in the trophoblasts. The results have also suggested that only a minority of *CRH* gene copies is configured epigenetically to be transcriptionally active in response to 8-Br-cAMP. The size of this subpopulation, potentially influenced by the uterine environment, may define the gestational trajectory of CRH expression in normal and pathological pregnancies.

## Materials and Methods

### Primary human trophoblast cell isolation and culture

The study was approved by the Hunter New England Health Human Ethics Committee and the University of Newcastle Human Ethics Committee (Approval No.: 03/02/12/3.15). All participants provided written informed consent to participate in the study. Placentae were obtained from uncomplicated singleton pregnancies after spontaneous labour and delivery at the John Hunter Hospital, Newcastle NSW, Australia. Participants delivered at term (37–41 weeks of pregnancy) as confirmed by early gestation ultrasound dating. Labour was uninduced and unaugmented, and no maternal or fetal morbidities were encountered during pregnancy and labour. Cytotrophoblasts were isolated, purified and cultured as described by Kliman et al., [4] with minor modifications detailed in the Supplementary Methods (S1 File). Trophoblast cultures were established from six placentae, referred to as Placentae No. 1 –No. 6 in chronological order of collection. *CRH* mRNA, CRH peptide and *CRH* promoter methylation were determined in cultures from Placentae No. 1, No. 2 and No. 3. Chromatin isolated from one primary trophoblast culture was sufficient to perform 5 combined chromatin immunoprecipitation (ChIP)–bisulfite-sequencing procedures. For this reason, cultures from Placentae No. 1, No. 2 and no. 3 were used for pCREB, TBP (TATA-binding protein, after vehicle and 8-Br-cAMP treatment) and acetylated-histone 3 (acH3) ChIP-bisulfite sequencing, and Placentae No. 4, No. 5 and No. 6 were used for acetylated histone 4 (acH4), trimethyl histone 3-lysine 4 (H3K4me3), trimethyl histone 3-lysine 27 (H3K27me3) and trimethyl histone 3-lysine 9 (H3K9me3) ChIP-bisulfite sequencing. Cultures from all 6 placentae were processed for RNA polymerase II (Pol-II) ChIP-bisulfite sequencing analysis. The placenta numbers are indicated in the respective figures.

### RNA extraction, cDNA synthesis and real-time PCR

The procedures of extracting and purifying RNA from cultured trophoblasts, removing contaminating DNA, reverse transcription and measuring *CRH* mRNA relative abundance has been described in detail [16] including primers [17] (S3 Table), reference RNA choice (Alien) and quantitation method (delta-delta-Ct) [18].

### Measurement of CRH peptide concentration

CRH peptide concentration in the culture media was determined with a validated radioimmunoassay as described [1, 19].

### Clonal bisulfite sequencing of the CRH proximal promoter

The methods of DNA extraction, bisulfite conversion, amplification and isolation of the *CRH* proximal promoter fragment, cloning and sequencing have been described in detail [16] and outlined in Supplementary Methods (S1 File).

### Chromatin immunoprecipitation (ChIP)

The previously described ChIP procedure [20, 21] has been adapted to cultured trophoblast cells, as detailed in Supplementary Methods (S1 File).

### Bisulfite sequencing of ChIP-selected DNA

Purified DNA recovered after ChIP was bisulfite-converted using the Cells-to-CpG Bisulfite Conversion Kit (Applied Biosystems) with thermal cycle conditions following the manufacturer’s protocol for small input DNA (between 50 pg and 100 ng). The *CRH* proximal promoter sequence was amplified by nested PCR, gel purified, and processed for clonal bisulfite sequencing as described in Ref. [22] and Supplementary Methods (S1 File).

### Data analysis

#### CRH mRNA relative abundance and peptide production

The *CRH* mRNA relative abundance and peptide production values were logarithmically transformed to approach normal distribution and analysed by ANOVA (analysis of variance) with time (0, 24, 48 and 72 hours), treatments (± 8-Br-cAMP), time-treatments interaction and placenta ID as independent variables. Time, treatments and their interaction were entered in the model as repeated measures variables. Variance on placenta ID was significant. The ANOVA was followed by postestimation where contrasts were tested between time and treatment at each experimental time point by F-tests. The P-values were corrected for multiple testing according to Sidak.

#### CRH promoter methylation

Methylation was considered a binary dependent variable at each CpG site being either unmethylated or methylated. Data were entered in a generalized estimating equations (GEE) model using the logit link function as recommended for repeated measures analysis for binary outcomes [23]. Placenta number, representing independent experimental repeats, was entered as the panel variable. Postestimation Wald tests were used to determine the overall significance of the effects associated with treatment and CpG sites. The association between treatments and epiallele frequencies was tested using Pearson’s chi-square test.

#### Chromatin immunoprecipitation

**Fig 1:**
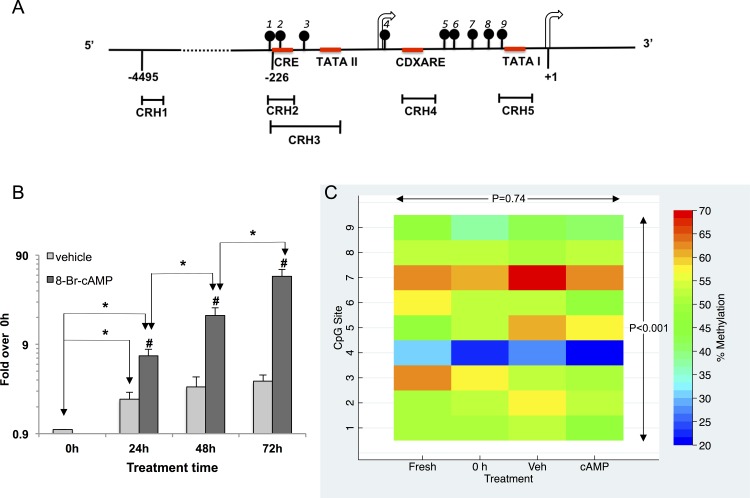
*CRH* promoter structure, cAMP-stimulation and promoter methylation in primary human trophoblasts. (A) Scheme of the *CRH* promoter. The arrows flag the two principal transcription initiation sites. CpG sites are marked by the numbered lollypop symbols, and red lines denote regulatory sequences. Promoter regions amplified by real-time PCR after chromatin immunoprecipitation (CRH1–5) are also indicated. The first transcribed nucleotide from the proximal initiation site is +1. (B) *CRH* mRNA relative abundance in trophoblast cultures. Cells were treated with vehicle or 8-Br-cAMP for the indicated times and mRNA relative abundance was determined by real-time RT-PCR. Bars show the average and SEM of 3 independent experiments as fold-increase over 0 h. *, significant pairwise differences between the indicated time points; #, significant stimulation by 8-Br-cAMP at each time point (P < 0.05, ANOVA with repeated measures). (C) Heat map demonstrating CpG methylation levels in the *CRH* proximal promoter. Methylation levels are illustrated by the color scale on the right. The vertical axis shows the numbers of the CpG sites and the horizontal axis indicates the treatments, which included freshly isolated cells (Fresh), adherent cells after 16 h in culture (0 h), vehicle treatment (Veh) or 8-Br-cAMP treatment (cAMP) for the following 72 h. Methylation levels were significantly different among CpG sites (P<0.001, but not among treatments, GEE-Generalized estimating equations).

ChIP data, expressed as per cent recovery of input, were transformed to achieve normal distribution and analysed by repeated measures ANOVA with the following independent variables: *CRH* promoter region (Sites 1–5, Fig 1A), time_treatment (0h, 72 h vehicle, 72 h 8-Br-cAMP), interaction between time_treatment and Sites. Placenta number was entered in the model representing experimental repeats. Postestimation F-tests were performed to explore time_treatment effects at each promoter site. The P-values were adjusted for multiple testing according to Sidak.

#### Other statistical procedures

Methylation frequencies between ChIP-selected and unselected promoter epialleles (shown in S2 Table) were compared by Fisher’s exact test. The incidences of epialleles bearing methylation patterns compatible with Pol-II or pCREB binding (Table 1) were compared using the Exact Unconditional Homogeneity/Independence Test [24] (accessed at www.stat.ncsu.edu/exact). Data obtained with different placentae were pooled for these tests. Correlations were established using Spearman’s non-parametric method. All statistical calculations (except for the Berger test) were performed using the STATA software package (College Station, TX USA). P-values < 0.05 were considered significant.

**Table 1:** Incidence of *CRH* promoter epialleles compatible with Pol-II and pCREB binding in trophoblasts.

DNA Source	Number of promoter copies sequenced	Number of epialleles compatible with Pol-II binding	% (Pol-II)	Number of epialleles compatible with pCREB binding	% (pCREB)
**Unselected DNA from**					
Fresh cells	30	1	3.3	0	0
Untreated cells (0 h)	30	1	3.3^**a**^	0	0
Vehicle-treated cells	30	0	0^**a**^	0	0
8-Br-cAMP-treated cells	30	0	0	0	0
**Pol-II-selected DNA from**					
8-Br-cAMP-treated cells	60	60	100	0	0
**pCREB-selected DNA from**					
8-Br-cAMP-treated cells	30	0	0	30	100
**TBP-selected DNA from**					
Vehicle-treated cells	29	15	51.7^**#**^^**,b**^	4	13.8^**&**^^**,c**^
8-Br-cAMP-treated cells	31	14	45.2^**$**^^**,b**^	7	22.6*^,c^
**Acetyl-H3-selected DNA from**					
8-Br-cAMP-treated cells	30	22	73.3^**$**^	8	26.7*
**Acetyl-H4-selected DNA from**					
8-Br-cAMP-treated cells	29	0	0	0	0
**H3K4me3-selected DNA from**					
8-Br-cAMP-treated cells	30	0	0	0	0
**H3K27me3-selected DNA from**					
8-Br-cAMP-treated cells	30	2	6.7^**$**^	6	20*
**H3K9me3-selected DNA from**					
8-Br-cAMP-treated cells	28	1	3.6	0	0

Primary cytotrophoblast cultures were treated with vehicle or 8-Br-cAMP (2.5x10^-4^ moles/L) for 72 h. ^**a**^ *vs*. ^**a**^, ^**b**^ *vs*. ^**b**^ and ^**c**^ *vs*. ^c^ are not significantly different.

^**#**^ and ^**$**^, incidences of Pol-II compatible epialleles are significantly different in unselected DNA from vehicle-treated and 8-Br-cAMP-treated cells, respectively

^**&**^ and *, incidences of pCREB compatible epialleles are significantly different in unselected DNA from vehicle-treated and 8-Br-cAMP-treated cells, respectively. Exact Unconditional Homogeneity/Independence Tests were performed at www.stat.ncsu.edu/exact/.

## Results

### Time course of *CRH* mRNA induction and peptide production in trophoblast primary cultures and the effect of 8-Br-cAMP

Cytotrophoblast cells spontaneously syncytialised during 72 h in culture as reported previously [4, 25]. Syncytial differentiation was accompanied by significant 2.2–3.5- fold increase of *CRH* mRNA abundance between 24 h and 72 h of culture (Fig 1B). Treatment with 8-Br-cAMP (2.5 x 10^−4^ moles/L) stimulated *CRH* mRNA levels relative to vehicle at all time points, which was expected [5, 7, 22, 26–28] with a maximum of 15-fold increase at 72 h (P < 0.001). Thus, *CRH* mRNA abundance was 52-fold higher after 72 h of 8-Br-cAMP treatment than in untreated cells at 0 h showing robust time dependent upregulation of *CRH* gene activity (Fig 1B). CRH peptide release followed a similar trend. CRH peptide was detectable in the culture medium at 24 h, and its concentration increased significantly, 3.3-fold, between 24 h and 48 h and further 2.9-fold between 48 h and 72 h. 8-Br-cAMP significantly increased CRH peptide production compared to vehicle at each time point (S1 Fig). In line with these observations, *CRH* mRNA and peptide values measured under identical treatment conditions were highly correlated (Spearman’s rho = 0.86, P < 0.001; 21 observations).

### Methylation of CpG dinucleotides in the *CRH* proximal promoter

The *CRH* proximal promoter contains 9 CpG dinucleotides as illustrated in Fig 1A. Cytosine methylation at these sites was determined by clonal bisulfite sequencing. Ten randomly selected clones were sequenced at each treatment condition with three biological replicate cultures. The heat map in Fig 1C shows % methylation at each CpG in freshly isolated cytotrophoblasts (Fresh), after 16 h of culture (0 h treatment) and after treatment with vehicle (Veh) or 8-Br-cAMP (cAMP) for additional 72 h. We chose the 72 h treatment period because 8-Br-cAMP-induced gene activation was the strongest at this time. Overall methylation frequency varied between 47.8% and 51.1% exhibiting no significant difference with treatments or incubation times (9 CpGs x 10 clones x 3 placentae = 270 CpGs at each treatment). Methylation frequencies varied significantly, however, among the individual CpG sites (10 clones x 3 placentae x 4 treatments = 120 CpGs per site) with CpG4 being the lowest (25%) and CpG7 being the highest (64.2%) (Fig 1C). These results suggested that methylation of CpGs in the *CRH* promoter was partial and differential. Furthermore, methylation was stable under our experimental conditions with frequencies exhibiting no significant change during culture, with syncytial differentiation, or in response to 8-Br-cAMP.

**Fig 2:**
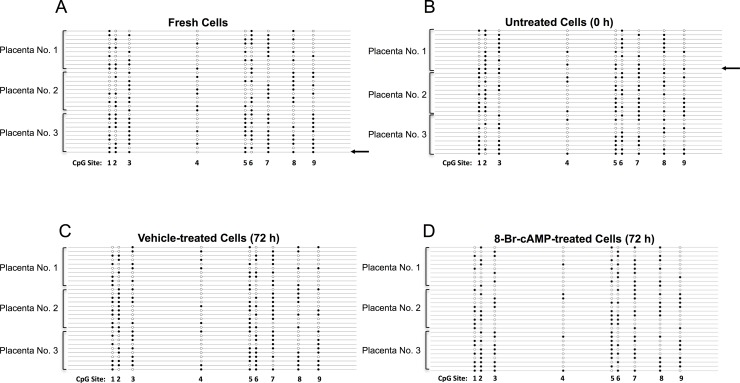
Methylation patterns of the *CRH* promoter. Patterns of CpG methylation in the *CRH* proximal promoter were determined by clonal bisulfite sequencing. Ten randomly selected clones representing individual copies of the promoter were sequenced under each treatment condition from cultures derived from 3 placentae. (A) Freshly isolated cells, (B) cells cultured for 16 h without treatment, (C) cells treated with vehicle or (D) with 8-Br-cAMP for the subsequent 72 h period. The horizontal lines indicate individual promoters, and open and closed circles denote unmethylated and methylated CpGs, respectively. CpG sites are numbered as in Fig 1A. The arrows (**←**) mark the two promoter copies with methylation patterns identical to a pattern found in the Pol-II-bound promoter fraction isolated by ChIP, shown in Fig 6B.

The methylation patterns of the cloned individual copies of the promoter are presented in Fig 2A–2D. Overall, 106 distinct methylation patterns (epialleles) were found in the four groups, and there was no significant association between treatments and epiallele frequencies (S1 Table). The methylation patterns did not indicate allele-specific methylation despite the overall methylation levels being close to 50%.

### Histone modifications at the *CRH* promoter

Posttranslational histone modifications alter chromatin structure and influence transcription factor binding to chromatin. We have used chromatin immunoprecipitation (ChIP) to determine the level of key histone modifications at the *CRH* promoter and explore their involvement in the epigenetic control of *CRH* expression. For the ChIP analyses, four PCR primer pairs (CRH2-5) were used to scan the proximal promoter, while CRH1 served as the upstream control. The positions of the amplicons are shown in Fig 1A. Accordingly, the CRE was covered by CRH2 and-3, the first and the second TATA-box (TATA I and TATA II) were covered by CRH5 and CRH3, respectively, and the alternative cAMP-response element comprising CDXARE (caudal-type homeobox protein response element) [28], was included in CRH4.

**Fig 3:**
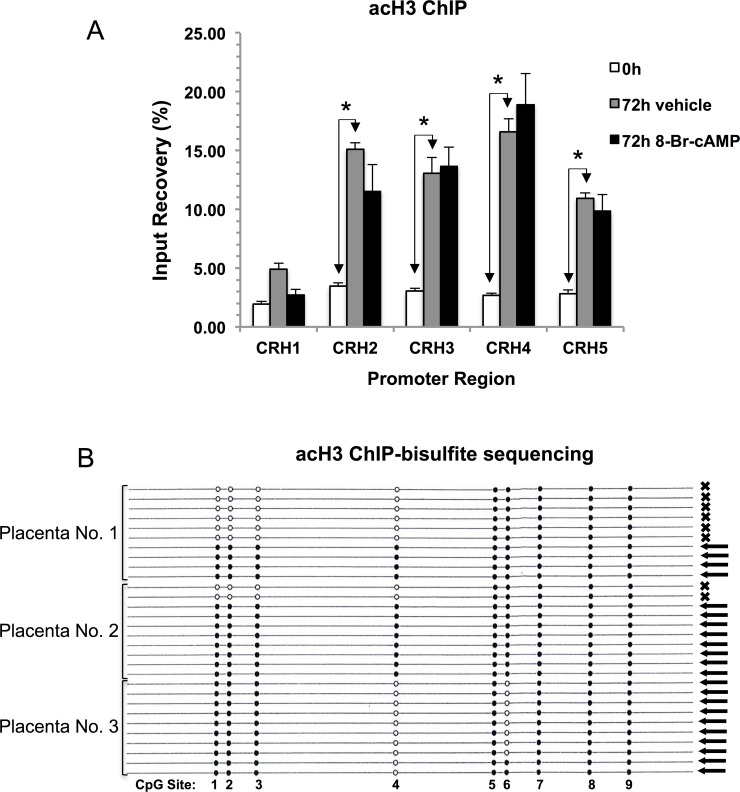
Methylation patterns of *CRH* promoter copies marked by histone-3 acetylation (acH3). (A) acH3 at the *CRH* proximal promoter was determined by ChIP using a (pan-)acetylated histone-3 antibody. Significant treatment effects at each promoter region (Fig 1A) are denoted by asterisk (*, P < 0.05, ANOVA with repeated measures, N = 3 placentae). (B) Methylation patterns of promoter copies associated with acH3 in 8-Br-cAMP-treated cultures. Arrows (←) mark promoter epialleles compatible with Pol-II binding, while (**x**) marks epialleles that can bind pCREB shown in Fig 6B and 6D, respectively. Placenta numbers are on the left. Filled circles indicate methylated CpGs and open circles indicate unmethylated CpGs. The positions of CpG Sites are as in Fig 1A.

**Fig 4:**
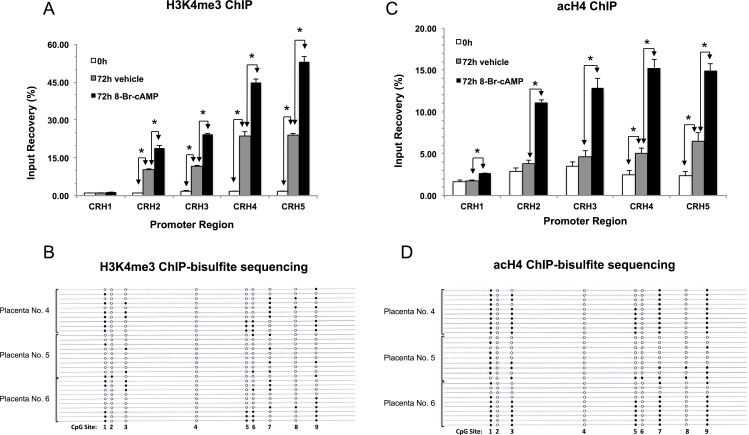
Methylation patterns of *CRH* promoter copies marked by histone-3, lysine-4 trimethylation (H3K4me3) and histone-4 acetylation (acH4). (A) H3K4me3 and (C) acH4 at the *CRH* proximal promoter were measured by ChIP. Significant treatment effects on modification levels are indicated by asterisks at each promoter region (Fig 1A; *, p < 0.05, ANOVA with repeated measures, N = 3 placentae). (B) and (D) Methylation patterns of promoter copies marked by H3K4me3 and acH4, respectively, were determined in 8-Br-cAMP-treated cultures. Placenta numbers are on the left; filled and open circles indicate methylated and unmethylated CpGs, respectively. The positions of CpG Sites are as shown in Fig 1A. Notably, none of the H3K4me3- or acH4-associated epialleles had methylation patterns compatible with Pol-II or pCREB binding.

The results of ChIP analysis using a (pan-)acetyl-histone-3 (acH3) antibody are shown in Fig 3A. Histone-3 acetylation is typically associated with transcriptionally active genes [29] and it has been detected in the *CRH* promoter area including the upstream control, CHR1. The level of acH3 increased significantly during 72h of culture in the region covered by CRH2 –CRH5. There was no additional increase in 8-Br-cAMP-treated cells. Histone-4 acetylation (acH4, measured by a (pan-)acetyl-histone-4 antibody) and histone-3, lysine-4 trimethylation (H3K4me3) are also associated in general with transcriptionally active genes [29, 30]. ChIP results in Fig 4A and 4C demonstrate that both modifications are present at the *CRH* promoter, increase significantly during culture and are enhanced further by 8-Br-cAMP treatment. We have also determined whether the repressive histone modifications histone-3, lysine 27-trimethylation (H3K27me3) and histone-3, lysine 9-trimethylation (H3K9me3) [29, 30] are present at the *CRH* proximal promoter. Both modifications were found throughout the ChIP-scanned region (including the upstream control, S2 Fig Panel A and S3 Fig Panel A, respectively). The levels of these inhibitory modifications dropped significantly during culture and even further in the presence of 8-Br-cAMP (with the exception of H3K9me3 at CRH5, showing increase (S3 Fig Panel A).

### Transcription factor binding to the *CRH* promoter

**Fig 5:**
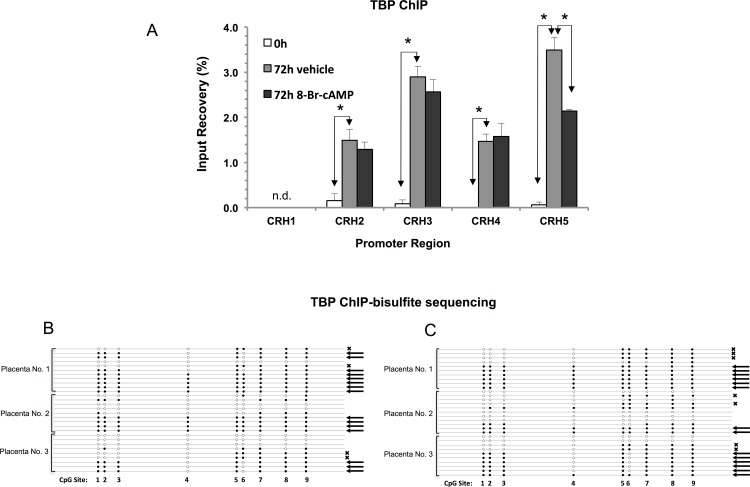
Methylation patterns of *CRH* promoter copies bound by TATA-binding protein (TBP). (A) Binding of TBP to the *CRH* proximal promoter was determined by ChIP. Significant treatment effects at each promoter region (shown in Fig 1A) are indicated by asterisks (*, P < 0.05, ANOVA with repeated measures, N = 3 placentae; n.d, undetectable ChIP signal). (B) and (C), Methylation patterns of promoter copies bound by TBP in vehicle treated and 8-Br-cAMP-treated cultures, respectively. Arrows (←) on the right indicate patterns compatible with Pol-II binding, while (**x**) denotes methylation pattern compatible with binding by pCREB as shown in Fig 6B and 6D, respectively. Placenta numbers are on the left; filled circles indicate methylated CpGs and open circles indicate unmethylated CpGs. The positions of CpG Sites are as shown in Fig 1A.

The *CRH* proximal promoter contains two TATA box sequence elements upstream of the two major transcription initiation sites, respectively (Fig 1A). In transcriptionally active promoters these elements are expected to associate with the TATA-binding protein TBP, a general transcription factor that is part of the preinitiation complex [31]. ChIP results in Fig 5A demonstrate that TBP-binding to the promoter was low in untreated cells and increased significantly over both TATA sites in cultures treated with vehicle for 72h. Stimulation with 8-Br-cAMP during the 72h culture period did not result in further increase in TBP binding, just a partial decrease of the ChIP signal over the region covered by CRH5. (The reason for this decrease is uncertain, but may be technical such as epitope masking [32].)

**Fig 6:**
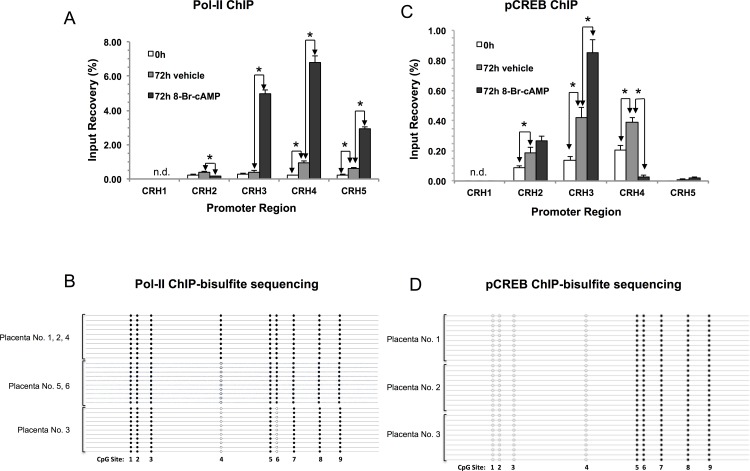
Methylation patterns of the *CRH* promoter bound by RNA-polymerase 2 (Pol-II) and phospho-cAMP response element-binding protein (pCREB). (A) Pol-II binding and (C) pCREB binding to the *CRH* promoter were determined by ChIP. PCR-amplified promoter regions are shown in Fig 1A. Bars represent means ± SEM (N = 6 and 3 placentae for Pol-II and pCREB, respectively). Asterisks indicate significant pairwise differences between treatments at each region (*, P < 0.05, ANOVA with repeated measures). Binding to the CRH1 region was undetectable (n.d.). (B) and (D), methylation patterns of *CRH* promoter copies bound by Pol-II and pCREB, respectively, were determined by clonal bisulfite sequencing of DNA isolated by ChIP. Ten randomly selected clones generated from 8-Br-cAMP treated cultures from each placenta were sequenced. Placenta numbers are on the left; filled and open circles indicate methylated and unmethylated CpGs, respectively. CpG Sites numbers are as shown in Fig 1A.

Binding of the transcription factor pCREB to the CRE in the *CRH* promoter is considered critical for *CRH* gene regulation in trophoblast cells [33]. To determine pCREB binding to the *CRH* promoter in our culture system we performed ChIP analysis using an antibody specific for this transcription factor. Fig 6C shows that pCREB binding to the region scanned by CRH2–4 was detected in untreated cells (0h). Binding of pCREB to this region increased significantly during culture for 72h. Treatment with 8-Br-cAMP significantly increased pCREB binding to the CRH3 region and concomitantly decreased pCREB binding at the CRH4 region enhancing the ChIP- signal at the CRE.

To assess the transcriptional activity of the *CRH* gene, we have performed chromatin immunoprecipitation (ChIP) using an antibody against RNA-polymerase-II (Pol-II; Fig 6A). Small, but significant binding of Pol-II was detected to the CRH4-5 region after 72 h of culture compared to 0 h in agreement with the increase of *CRH* mRNA level during spontaneous syncytialisation (Fig 1B). Treatment with 8-Br-cAMP resulted in a strong increase of Pol-II binding to the promoter region scanned by CRH3-5, which was in agreement with the robust stimulation of mRNA expression by the cyclic nucleotide.

### The relationship of CpG methylation with transcription factor binding and histone modifications at the *CRH* promoter

The diverse patterns of methylation (Fig 2, S1 Table) indicated that a variety of *CRH* promoter epialleles was present in the trophoblast cell population. An important question to address is whether the various epialleles are equivalent functionally or show differences of ability to harbor histone modifications and drive gene expression during syncytial differentiation and cAMP-stimulation. Pol-II ChIP with 8-Br-cAMP-treated cells yielded sufficient amounts of DNA to perform bisulfite sequencing to assess methylation levels and epiallele variety. Data in S2 Table show that the methylation level of CpGs in Pol-II-associated promoters was 92.3%, significantly higher than in the non-immunoprecipitated control (47.4%, p<0.001). Considering that CpG methylation levels did not change under our experimental conditions (Fig 1B), this indicated that the *CRH* gene copies bound by Pol-II had relatively high levels of promoter methylation. Furthermore, examination of the arrangement of methylated CpGs in individual cloned copies (Fig 6B) revealed that the methylation pattern of Pol-II-associated promoters was uniform in each placenta (N = 6) and followed 3 distinct types: all 9 CpGs methylated (3 placentae), all but CpG4 methylated (2 placentae) and all but CpGs4 and 6 methylated. Notably, the CRE (containing CpG2) was methylated in all copies associated with Pol-II suggesting that its pCREB-binding activity essential for mediating 8-Br-cAMP-stimulation [34] was suppressed.

Bisulfite sequencing of promoter copies immunoprecipitated with pCREB antibody indicated that he pattern of CpG methylation was uniform within and among placentae (N = 3 placentae with 10 promoter copies each) with CpGs 1, 2, 3, and 4 unmethylated and CpGs 5, 6, 7, 8, and 9 methylated (Fig 6D). Thus, CpG2 in the CRE was unmethylated as expected in agreement with pCREB binding [34].

*CRH* promoter DNA immunoprecipitated by the TBP antibody from vehicle treated and 8-Br-cAMP treated cultures was bisulfite sequenced as well. The TBP-bound *CRH* promoters had significantly higher methylation levels than promoter sequences in control (not ChIP-selected) DNA in both vehicle treated and 8-Br-cAMP treated cells (S2 Table). In addition, promoter copies associated with TBP were significantly enriched in methylation patterns compatible with Pol-II and pCREB binding (Fig 5B and 5C and Table 1).

We have performed ChIP-bisulfite analyses to explore whether histone modifications associate differentially with *CRH* promoter epialleles. Bisulfite sequencing of the DNA precipitated with the acH3 antibody indicated significantly higher methylation level of the *CRH* promoters than in the non-immunoprecipitated control (S2 Table). The methylation patterns of all sequenced promoter copies were compatible with either Pol-II or pCREB binding (Fig 3B and Table 1). Thus, histone-3 acetylation not only increased during syncytial differentiation, but it appeared selective at *CRH* gene copies targeted by Pol-II and pCREB in 8-Br-cAMP-treated cells.

The methylation level of *CRH* promoter copies associated with acH4 and H3K4me3 modifications, determined by ChIP-bisulfite sequencing using a (pan-)acH4 and a H3K4me3 antibody with 8-Br-cAMP-treated cells, was significantly lower than the non-immunoprecipitated control (S2 Table). Furthermore, the methylation patterns of the cloned promoter copies indicated that these histone modifications were not associated with promoter epialleles that bound either Pol-II, pCREB, or were acetylated at H3 (Table 1 and Fig 4B and 4D *vs*. Figs 6B, 6D and 3B).

The overall methylation level of the H3K27me3- associated promoters was not different from the non-immunoprecipitated control (S2 Table), but this group of promoter copies was enriched significantly in Pol-II and pCREB-binding epialleles (S2 Fig Panel A and Table 1). The H3K9me3-associated promoter copies were less methylated than the non-immunoprecipitated control and were not enriched in epialleles binding Pol-II or pCREB (S3 Fig Panel B, S2 Table and Table 1).

## Discussion

The results of this study show that *CRH* expression is controlled in trophoblasts by epigenetic mechanisms that include histone modifications and DNA methylation. Syncytial differentiation in primary cultures was accompanied by increasing levels of activating (acH3, acH4, H3K4me3) and decreasing levels of suppressive (H3K27me3, H3K9me3) histone modifications at the *CRH* proximal promoter. Treatment with 8-Br-cAMP robustly stimulated *CRH* gene expression and augmented the changes in histone modifications with the exception of acH3, which appeared unresponsive to cAMP (Fig 3A). This feature suggests that H3-acetylation may be a cAMP-independent process poising the *CRH* promoter for increased transcription factor access during differentiation. In agreement with this, enhanced recruitment of TBP and pCREB was observed to the promoter during syncytialisation in the absence of 8-Br-cAMP (Figs 5A and 6C). Treatment with the cyclic nucleotide caused a marked increase of Pol-II recruitment to the poised promoter in agreement with the large increase of *CRH* gene activity (Fig 6A).

The analysis of CpG methylation radically reshaped the picture of epigenetic *CRH* gene regulation based solely on ChIP data. Bisulfite sequencing has demonstrated that the *CRH* proximal promoter, comprising 9 CpG dinucleotides, is partially methylated in trophoblast cells. Methylation level overall was close to 50% exhibiting no significant change during culture, or after exposure to 8-Br-cAMP. Methylation levels of the particular CpG sites were significantly different, however, indicating context specific susceptibility to methylation, which remained unchanged under the culture conditions employed. The patterns of methylation determined by clonal bisulfite sequencing did not indicate allele dependency (such as a mixture of either fully methylated or unmethylated promoter copies); rather, they revealed that a variety of epialleles is present in the trophoblast cell population. These attributes suggest that the CRH proximal promoter represents an “intermediate methylation region” (IM) in the genome as determined by recent comparative epigenomic studies involving a number of human tissues and cell types [35]. The IM regions are generally associated with tissue specific regulatory functions [35] in line with the observation that promoter methylation controls hypothalamic *CRH* expression in rodents [10, 36].

The varying methylation patterns of individual promoter copies raised the question whether the *CRH* promoter epialleles were functionally different. We addressed this possibility using ChIP combined with bisulfite sequencing of the immunoprecipitated DNA [37], which is an novel approach to explore cross-talk between transcription factor binding, histone modifications and DNA methylation at both the genomic and the single gene levels [38, 39]. Surprisingly, Pol-II-associated *CRH* promoter copies were significantly more methylated than the promoter population overall, and only 3 specific methylation patterns were detected in the Pol-II-bound fraction: (1) all 9 CpGs methylated, (2) only CpG4 unmethylated and (3) CpGs 4 and 6 unmethylated. During the study we have encountered 117 various *CRH* promoter methylation patterns in the primary trophoblasts (106 in Fig 2, listed in S1 Table, and additional 11 in the other experimental settings). Finding only three of them in the Pol-II ChIP-isolated fractions indicates high epiallele selectivity of Pol-II binding. A further striking observation was that CpG2 in the CRE was uniformly methylated in the Pol-II-bound fraction. This argues against the involvement of the pCREB-CRE interaction mediating the stimulation of *CRH* gene activity by 8-Br-cAMP, since this interaction is disrupted by CpG methylation within the CRE [34]. Nevertheless, pCREB binding increased to the CRE region during syncytialisation and cAMP-stimulation, and the pCREB-bound promoters were unmethylated at CpG2, as expected. The methylation pattern of the pCREB-bound promoter copies was different from the patterns bound by Pol-II, however, demonstrating that Pol-II and pCREB bound to separate sub-populations of the CRH promoter characterized by distinct epialleles.

ChIP-bisulfite analysis of TBP binding showed significant enrichment of promoter epialleles compatible with Pol-II and pCREB binding, which suggested that chromatin remodeling that allowed promoter access for TBP in syncytia occurred preferentially in a subpopulation of *CRH* epialleles that were capable of binding Pol-II and pCREB.

We have extended the ChIP-bisulfite analysis to key histone modifications that contribute to the epigenetic landscape, influence chromatin structure and correlate with gene activity [40]. Remarkably, the promoter fraction associated with acetylated histone-3 consisted entirely of copies possessing methylation patterns compatible with either Pol-II or pCREB binding, which highlights the importance of this histone modification for transcription factor access to the region. Dynamic acetylation of H3K9 has recently been found critical for the gestational and glucocorticoid dependent upregulation of *CRH* expression in trophoblasts and for transcription factor binding to *CRH* gene regulatory elements [12]. We used a pan-K-acetylated H3 antibody in our ChIP assays, but the results by Di Stefano et al. [12] suggest that the immunoprecipitated chromatin fraction we have analysed for DNA methylation contained acetyl-H3K9. It remains to be established whether a dynamic turnover of this modification occurs when *CRH* expression is stimulated by 8-Br-cAMP.

Promoter copies associated with the gene activating acH4 and H3K4me3 modifications did not carry CpG methylation patterns compatible with Pol-II and pCREB binding. This suggests that these modifications affected preferentially a subpopulation of *CRH* promoters not involved in the 8-Br-cAMP-evoked response. Enrichment of Pol-II- and pCREB-binding methylation patterns was significant in the H3K27me3-marked epialleles relative to the non-immunoprecipitated input suggesting that loss of this mark might have contributed to the epigenetic activation of *CRH* in the trophoblast cultures. Promoter copies marked by H3K9me3 did not show significant enrichment of Pol-II- and pCREB-binding epialleles arguing against the possibility that loss of this modification contributed appreciably to the changes in *CRH* gene expression under the conditions employed.

An important outcome of our ChIP-bisulfite analyses is that it has revealed the epigenetic, functional and structural heterogeneity of the *CRH* promoter population in term trophoblasts. Based on the epiallele selective associations between the *CRH* promoter, Pol-II and pCREB, we can infer with reasonable confidence that the CRE-pCREB interaction is not involved in the cAMP-dependent regulation of the gene, at least in our regulatory model using 8-Br-cAMP. Interestingly, this interaction was shown to be crucial for regulating *CRH* promoter activity in assays using transfected promoter-reporter constructs [41]. Our results suggest that 8-Br-cAMP induces the chromatin-integrated endogenous gene *via* a different pathway possibly involving the alternative cAMP-response element, which does not bind pCREB [7] (Fig 1A). The activating epigenetic marks H3K4me3 and acH4 are not required for the stimulation, but acH3 appears critical. The molecular apparatus that forms this unique signaling pathway remains to be determined.

A published quantitative analysis of Pol-II localisation in placental tissue has shown that 20–40% of syncytiotrophoblast nuclei are transcriptionally inactive [42]. Our ChIP-bisulfite analyses indicated that less than 5% of *CRH* promoter copies were engaged in transcription in 8-Br-cAMP-stimulated primary trophoblasts as only 2 of 120 sequenced *CRH* promoter copies showed methylation patterns compatible with Pol-II binding in DNA not subjected to ChIP (Fig 2). Epipolymorphism is widespread in the placenta [43], but the epiallele selectivity of *CRH* gene activation is intriguing even if more transcriptionally active *CRH* epialleles could be found in addition to the 3 we have identified by examining a larger number of placentae. It is unclear whether specific methylation patterns are involved functionally in the recruitment of particular *CRH* promoter copies for transcription, or just mark the selected group of epialleles. A related important question is whether other stimulants of *CRH* expression, such as glucocorticoids, target the same set of epialleles as 8-Br-cAMP. This is likely, since these epialleles are preferentially acetylated on histone-3, which is a hallmark of glucocorticoid action on *CRH* as reported [12]. In our hands glucocorticoid was a weak stimulant (eliciting less than 2-fold increase in *CRH* expression), not providing enough DNA for bisulfite sequencing after Pol-II ChIP. The function of the *CRH* promoter sub-population activated by H3K4me3 and acH4 modifications, but not involved in the 8-Br-cAMP-response, also remains to be determined. Finally, it will be critical to explore how is the epiallele distribution of *CRH* promoters established in the trophoblast cell population. Methylated CpG levels were unchanging with no indication of significant turnover in our culture system, which contained non-dividing term trophoblasts (Fig 1C). The trophoblast cells were isolated from term placentae after spontaneous labor, and the methylation levels and patterns could have been influenced by intrauterine conditions during pregnancy and in labor resulting in changes in the number of transcriptionally competent *CRH* epialleles. Data describing the epiallele distribution of the *CRH* promoter population in placentae at different times of gestation and after labor may uncover factors that impact on methylation levels and patterns and determine the gestational trajectory of CRH production in normal and pathological pregnancies.

## Supporting Information

S1 Fig CRH peptide production by primary trophoblast cell cultures. Cell cultures were treated with vehicle or 8-Br-cAMP (250 μM) and CRH peptide concentration in the culture medium was determined by radioimmunoassay. Bars show the average and SEM of 3 independent experiments. *, significant pairwise differences between time points as indicated by the arrows; #, significant stimulation by 8-Br-cAMP at each time point (p<0.05).
(PDF)

S2 Fig Methylation patterns of *CRH* promoter copies marked by histone-3 lysine-27 trimethylation (H3K27me3). (A) H3K27me3 marking at the *CRH* proximal promoter was determined by chromatin immunoprecipitation (ChIP). Significant treatment effects at each promoter region (Fig 1A) are denoted by asterisk (*, p<0.05, ANOVA with repeated measures, N = 3 placentae). (B) Methylation patterns of *CRH* promoter copies marked by H2K27me3 were determined by bisulfite sequencing of ChIP-isolated DNA from 8-Br-cAMP-treated cultures. Arrows (←) mark promoter epialleles compatible with Pol-II binding, while (**x**) indicates epialleles that can bind pCREB. Placenta numbers are on the left; filled and open circles indicate methylated and unmethylated CpGs, respectively. The positions of CpG Sites are as shown in Fig 1A.
(PDF)

S3 Fig Methylation patterns of *CRH* promoter copies marked by histone-3 lysine-9 trimethylation (H3K9me3). (A) H3K9me3 marking of the promoter was determined by chromatin immunoprecipitation (ChIP). Significant treatment effects at each promoter region (Fig 1A) are denoted by asterisk (*, p<0.05, ANOVA with repeated measures, N = 3 placentae). (B) Methylation patterns of *CRH* promoter copies in chromatin marked by H3K9me3 were determined by bisulfite sequencing of DNA isolated by ChIP from 8-Br-cAMP-treated cultures. The arrow (←) marks a promoter epiallele capable of Pol-II binding. Placenta numbers are on the left. Filled and open circles indicate methylated and unmethylated CpGs, respectively. The positions of CpG Sites are as shown in Fig 1A.
(PDF)

S1 Table Frequency of methylation patterns of the *CRH* promoter. (PDF)

S2 Table Methylation levels in *CRH* promoter copies associated with transcription factors and modified histones. (PDF)

S3 Table Primer Sequences. (PDF)

S1 File Supplementary Methods. (PDF)
