# Supplementary figures and images for: Promoter Methylation Pattern Controls Corticotropin Releasing Hormone Gene Activity in Human Trophoblasts
Source: PLoS One. 2017 Feb 2;12(2):e0170671. doi: 10.1371/journal.pone.0170671 (PMC5289476; doi:10.1371/journal.pone.0170671)

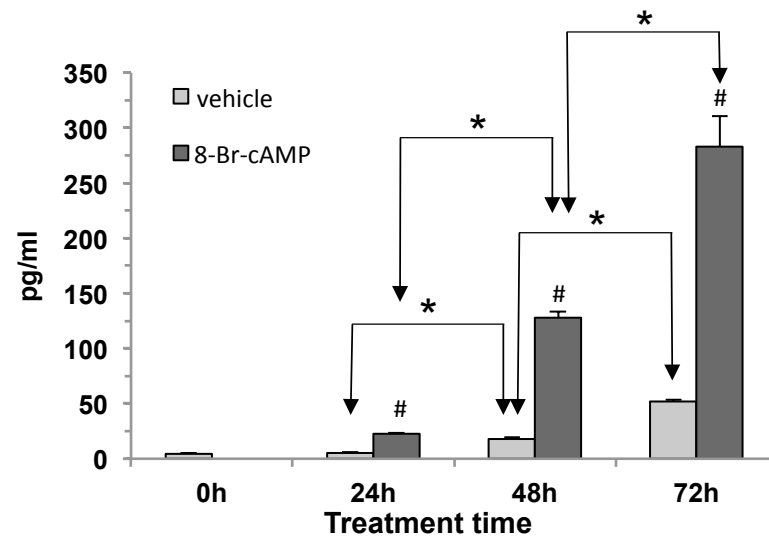

Supplement: S1 Fig — Cell cultures were treated with vehicle or 8-Br-cAMP (250 μM) and CRH peptide concentration in the culture medium was determined by radioimmunoassay. Bars show the average and SEM of 3 independent experiments. *, significant pairwise differences between time points as indicated by the arrows; #, significant stimulation by 8-Br-cAMP at each time point (p<0.05). (PDF) [file pone.0170671.s001.pdf]

A

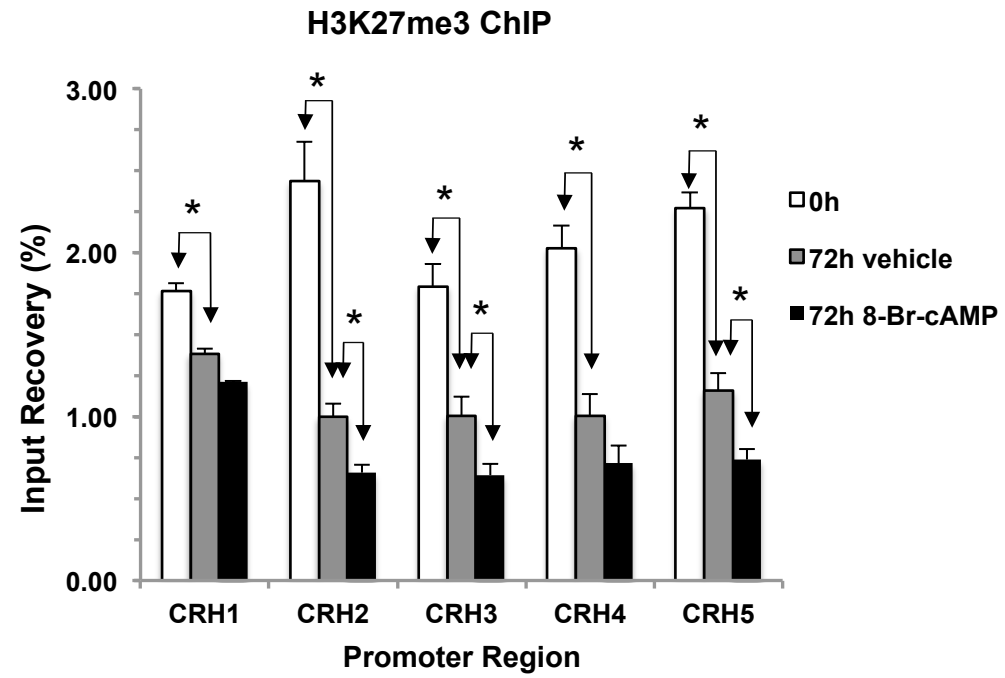

B

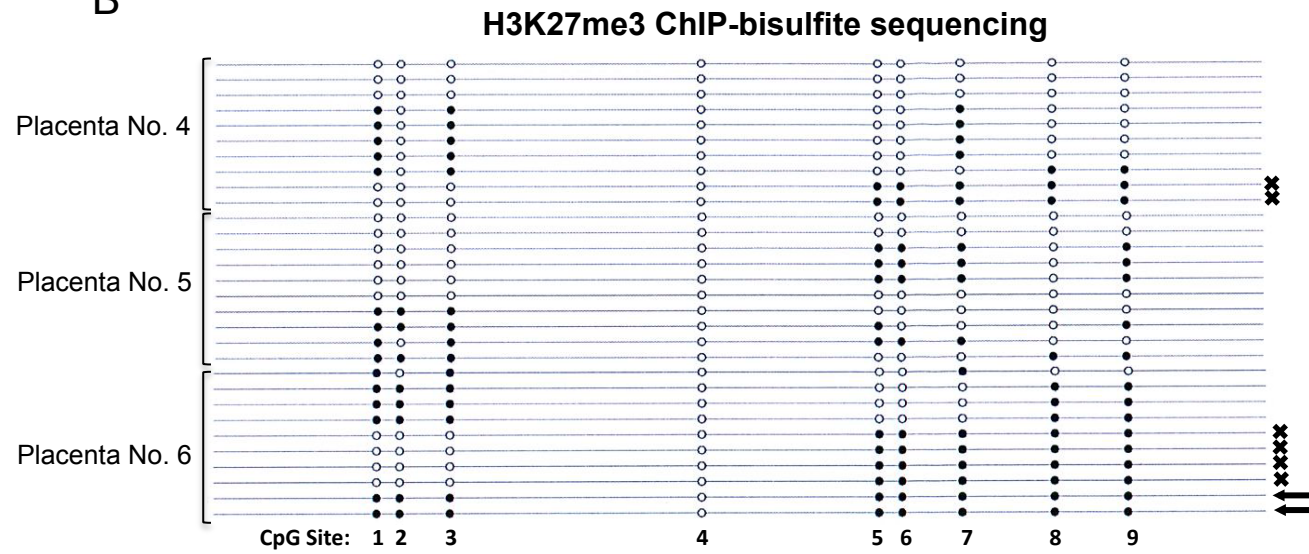

Supplement: S2 Fig — (A) H3K27me3 marking at the CRH proximal promoter was determined by chromatin immunoprecipitation (ChIP). Significant treatment effects at each promoter region (Fig 1A) are denoted by asterisk (*, p<0.05, ANOVA with repeated measures, N = 3 placentae). (B) Methylation patterns of CRH promoter copies marked by H2K27me3 were determined by bisulfite sequencing of ChIP-isolated DNA from 8-Br-cAMP-treated cultures. Arrows (←) mark promoter epialleles compatible with Pol-II binding, while (x) indicates epialleles that can bind pCREB. Placenta numbers are on the left; filled and open circles indicate methylated and unmethylated CpGs, respectively. The positions of CpG Sites are as shown in Fig 1A. (PDF) [file pone.0170671.s002.pdf]

A

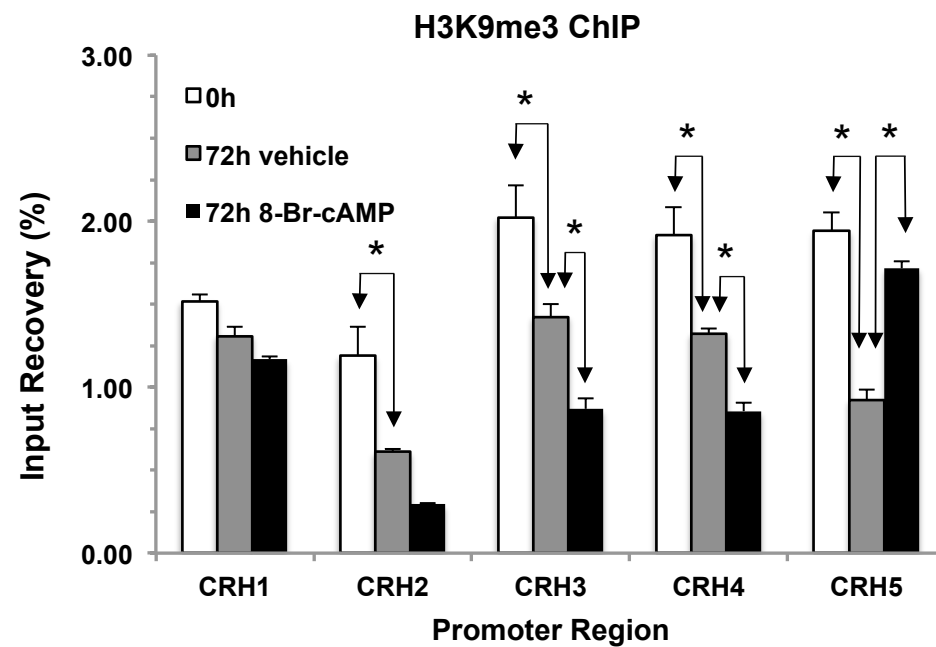

B

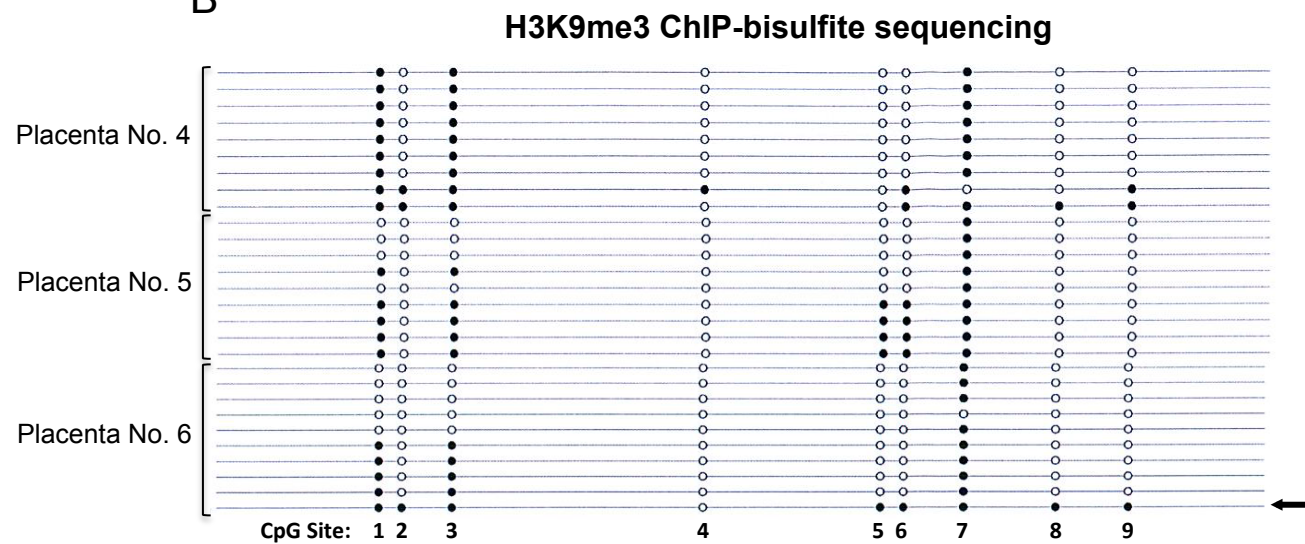

Supplement: S3 Fig — (A) H3K9me3 marking of the promoter was determined by chromatin immunoprecipitation (ChIP). Significant treatment effects at each promoter region (Fig 1A) are denoted by asterisk (*, p<0.05, ANOVA with repeated measures, N = 3 placentae). (B) Methylation patterns of CRH promoter copies in chromatin marked by H3K9me3 were determined by bisulfite sequencing of DNA isolated by ChIP from 8-Br-cAMP-treated cultures. The arrow (←) marks a promoter epiallele capable of Pol-II binding. Placenta numbers are on the left. Filled and open circles indicate methylated and unmethylated CpGs, respectively. The positions of CpG Sites are as shown in Fig 1A. (PDF) [file pone.0170671.s003.pdf]
