# Supplementary material for: Promoter Methylation Pattern Controls Corticotropin Releasing Hormone Gene Activity in Human Trophoblasts
Source: PLoS One. 2017 Feb 2;12(2):e0170671. doi: 10.1371/journal.pone.0170671 (PMC5289476; doi:10.1371/journal.pone.0170671)
Supplement: S1 Table — (PDF) [file pone.0170671.s004.pdf]

Supplementary Table 1
Frequency of methylation patterns of the CRH promoter

Treatments for pattern analysis: 1. Fresh; 2. 0h; 3. 72h with vehicle; 4. 72h with 8-Br-cAMP
Patterns are from 5' to 3' direction indicating u: unmethylated and m: methylated CpGs 1 - 9

Cell contents: frequency
expected frequency
chi2 contribution

pattern	Treatments for pattern analysis 1	2	3	4	Total
mumummmum	1	1	1	1	4
	1.0	1.0	1.0	1.0	4.0
	0.0	0.0	0.0	0.0	0.0
mmmumummm	2	1	0	0	3
	0.8	0.8	0.8	0.8	3.0
	2.1	0.1	0.8	0.8	3.7
mmmummmum	1	0	0	1	2
	0.5	0.5	0.5	0.5	2.0
	0.5	0.5	0.5	0.5	2.0

mmmummumu	1<br>0.5<br>0.5	0<br>0.5<br>0.5	1<br>0.5<br>0.5	0<br>0.5<br>0.5	2<br>2.0<br>2.0
mmmumumum	0<br>0.5<br>0.5	0<br>0.5<br>0.5	1<br>0.5<br>0.5	1<br>0.5<br>0.5	2<br>2.0<br>2.0
mumuummmm	0<br>0.5<br>0.5	0<br>0.5<br>0.5	2<br>0.5<br>4.5	0<br>0.5<br>0.5	2<br>2.0<br>6.0
muuuummuu	0<br>0.5<br>0.5	1<br>0.5<br>0.5	0<br>0.5<br>0.5	1<br>0.5<br>0.5	2<br>2.0<br>2.0
ummuummmu	1<br>0.5<br>0.5	1<br>0.5<br>0.5	0<br>0.5<br>0.5	0<br>0.5<br>0.5	2<br>2.0<br>2.0
umuuummmu	0<br>0.5<br>0.5	1<br>0.5<br>0.5	1<br>0.5<br>0.5	0<br>0.5<br>0.5	2<br>2.0<br>2.0
uumumuumu	1<br>0.5<br>0.5	1<br>0.5<br>0.5	0<br>0.5<br>0.5	0<br>0.5<br>0.5	2<br>2.0<br>2.0
uuuuummmu	0<br>0.5	1<br>0.5	0<br>0.5	1<br>0.5	2<br>2.0

	0.5	0.5	0.5	0.5	2.0
mmmmmmmuu	1<br>0.2<br>2.2	0<br>0.2<br>0.2	0<br>0.2<br>0.2	0<br>0.2<br>0.2	1<br>1.0<br>3.0
mmmmmummm	0<br>0.2<br>0.2	1<br>0.2<br>2.2	0<br>0.2<br>0.2	0<br>0.2<br>0.2	1<br>1.0<br>3.0
mmmmummuu	0<br>0.2<br>0.2	1<br>0.2<br>2.2	0<br>0.2<br>0.2	0<br>0.2<br>0.2	1<br>1.0<br>3.0
mmmmumumu	1<br>0.2<br>2.2	0<br>0.2<br>0.2	0<br>0.2<br>0.2	0<br>0.2<br>0.2	1<br>1.0<br>3.0
mmmmumuuu	0<br>0.2<br>0.2	0<br>0.2<br>0.2	0<br>0.2<br>0.2	1<br>0.2<br>2.2	1<br>1.0<br>3.0
mmmummmuu	1<br>0.2<br>2.2	0<br>0.2<br>0.2	0<br>0.2<br>0.2	0<br>0.2<br>0.2	1<br>1.0<br>3.0
mmmummuuu	0<br>0.2<br>0.2	0<br>0.2<br>0.2	1<br>0.2<br>2.2	0<br>0.2<br>0.2	1<br>1.0<br>3.0
mmmumuumm	0	0	0	1	1

	0.2	0.2	0.2	0.2	1.0
	0.2	0.2	0.2	2.2	3.0
mmmuummmm	1	0	0	0	1
	0.2	0.2	0.2	0.2	1.0
	2.2	0.2	0.2	0.2	3.0
mmmuumumm	0	1	0	0	1
	0.2	0.2	0.2	0.2	1.0
	0.2	2.2	0.2	0.2	3.0
mmmuuummu	0	0	0	1	1
	0.2	0.2	0.2	0.2	1.0
	0.2	0.2	0.2	2.2	3.0
mmmuuuuuu	0	1	0	0	1
	0.2	0.2	0.2	0.2	1.0
	0.2	2.2	0.2	0.2	3.0
mmummmmmu	0	1	0	0	1
	0.2	0.2	0.2	0.2	1.0
	0.2	2.2	0.2	0.2	3.0
mmummmmum	0	1	0	0	1
	0.2	0.2	0.2	0.2	1.0
	0.2	2.2	0.2	0.2	3.0
mmumummmu	0	0	0	1	1
	0.2	0.2	0.2	0.2	1.0
	0.2	0.2	0.2	2.2	3.0

mmumuuuum	0<br>0.2<br>0.2	0<br>0.2<br>0.2	1<br>0.2<br>2.2	0<br>0.2<br>0.2	1<br>1.0<br>3.0
mmuummumu	0<br>0.2<br>0.2	0<br>0.2<br>0.2	1<br>0.2<br>2.2	0<br>0.2<br>0.2	1<br>1.0<br>3.0
mmuummuuu	0<br>0.2<br>0.2	1<br>0.2<br>2.2	0<br>0.2<br>0.2	0<br>0.2<br>0.2	1<br>1.0<br>3.0
mmuumummm	0<br>0.2<br>0.2	1<br>0.2<br>2.2	0<br>0.2<br>0.2	0<br>0.2<br>0.2	1<br>1.0<br>3.0
mmuumummu	0<br>0.2<br>0.2	0<br>0.2<br>0.2	1<br>0.2<br>2.2	0<br>0.2<br>0.2	1<br>1.0<br>3.0
mmuumumuu	0<br>0.2<br>0.2	0<br>0.2<br>0.2	1<br>0.2<br>2.2	0<br>0.2<br>0.2	1<br>1.0<br>3.0
mmuumuumm	0<br>0.2<br>0.2	0<br>0.2<br>0.2	0<br>0.2<br>0.2	1<br>0.2<br>2.2	1<br>1.0<br>3.0
mmuumuuum	0<br>0.2<br>0.2	0<br>0.2<br>0.2	1<br>0.2<br>2.2	0<br>0.2<br>0.2	1<br>1.0<br>3.0

mmuuuummu	0<br>0.2<br>0.2	0<br>0.2<br>0.2	0<br>0.2<br>0.2	1<br>0.2<br>2.2	1<br>1.0<br>3.0
mummmmmum	0<br>0.2<br>0.2	0<br>0.2<br>0.2	1<br>0.2<br>2.2	0<br>0.2<br>0.2	1<br>1.0<br>3.0
mummumumu	1<br>0.2<br>2.2	0<br>0.2<br>0.2	0<br>0.2<br>0.2	0<br>0.2<br>0.2	1<br>1.0<br>3.0
mummumuum	0<br>0.2<br>0.2	1<br>0.2<br>2.2	0<br>0.2<br>0.2	0<br>0.2<br>0.2	1<br>1.0<br>3.0
mumummmmm	1<br>0.2<br>2.2	0<br>0.2<br>0.2	0<br>0.2<br>0.2	0<br>0.2<br>0.2	1<br>1.0<br>3.0
mumummmuu	0<br>0.2<br>0.2	0<br>0.2<br>0.2	0<br>0.2<br>0.2	1<br>0.2<br>2.2	1<br>1.0<br>3.0
mumummuum	0<br>0.2<br>0.2	0<br>0.2<br>0.2	0<br>0.2<br>0.2	1<br>0.2<br>2.2	1<br>1.0<br>3.0
mumumuuum	0<br>0.2	1<br>0.2	0<br>0.2	0<br>0.2	1<br>1.0

	0.2	2.2	0.2	0.2	3.0
mumuummuu	0<br>0.2<br>0.2	0<br>0.2<br>0.2	1<br>0.2<br>2.2	0<br>0.2<br>0.2	1<br>1.0<br>3.0
mumuuumuu	0<br>0.2<br>0.2	1<br>0.2<br>2.2	0<br>0.2<br>0.2	0<br>0.2<br>0.2	1<br>1.0<br>3.0
muummmumu	0<br>0.2<br>0.2	0<br>0.2<br>0.2	1<br>0.2<br>2.2	0<br>0.2<br>0.2	1<br>1.0<br>3.0
muummummu	0<br>0.2<br>0.2	0<br>0.2<br>0.2	1<br>0.2<br>2.2	0<br>0.2<br>0.2	1<br>1.0<br>3.0
muummuuuu	0<br>0.2<br>0.2	0<br>0.2<br>0.2	1<br>0.2<br>2.2	0<br>0.2<br>0.2	1<br>1.0<br>3.0
muumuummu	1<br>0.2<br>2.2	0<br>0.2<br>0.2	0<br>0.2<br>0.2	0<br>0.2<br>0.2	1<br>1.0<br>3.0
muuumummu	0<br>0.2<br>0.2	0<br>0.2<br>0.2	0<br>0.2<br>0.2	1<br>0.2<br>2.2	1<br>1.0<br>3.0
muuumumuu	0	1	0	0	1

	0.2<br>0.2	0.2<br>2.2	0.2<br>0.2	0.2<br>0.2	1.0<br>3.0
muuumuumu	1<br>0.2<br>2.2	0<br>0.2<br>0.2	0<br>0.2<br>0.2	0<br>0.2<br>0.2	1<br>1.0<br>3.0
muuuumuuu	1<br>0.2<br>2.2	0<br>0.2<br>0.2	0<br>0.2<br>0.2	0<br>0.2<br>0.2	1<br>1.0<br>3.0
muuuuummu	0<br>0.2<br>0.2	0<br>0.2<br>0.2	0<br>0.2<br>0.2	1<br>0.2<br>2.2	1<br>1.0<br>3.0
muuuuuuuu	1<br>0.2<br>2.2	0<br>0.2<br>0.2	0<br>0.2<br>0.2	0<br>0.2<br>0.2	1<br>1.0<br>3.0
ummmummmu	1<br>0.2<br>2.2	0<br>0.2<br>0.2	0<br>0.2<br>0.2	0<br>0.2<br>0.2	1<br>1.0<br>3.0
ummmuummm	0<br>0.2<br>0.2	0<br>0.2<br>0.2	1<br>0.2<br>2.2	0<br>0.2<br>0.2	1<br>1.0<br>3.0
ummummmmm	0<br>0.2<br>0.2	0<br>0.2<br>0.2	0<br>0.2<br>0.2	1<br>0.2<br>2.2	1<br>1.0<br>3.0

ummummmmu	0<br>0.2<br>0.2	1<br>0.2<br>2.2	0<br>0.2<br>0.2	0<br>0.2<br>0.2	1<br>1.0<br>3.0
ummummmum	1<br>0.2<br>2.2	0<br>0.2<br>0.2	0<br>0.2<br>0.2	0<br>0.2<br>0.2	1<br>1.0<br>3.0
ummummmuu	0<br>0.2<br>0.2	0<br>0.2<br>0.2	1<br>0.2<br>2.2	0<br>0.2<br>0.2	1<br>1.0<br>3.0
ummummumm	0<br>0.2<br>0.2	0<br>0.2<br>0.2	1<br>0.2<br>2.2	0<br>0.2<br>0.2	1<br>1.0<br>3.0
ummumummu	0<br>0.2<br>0.2	0<br>0.2<br>0.2	1<br>0.2<br>2.2	0<br>0.2<br>0.2	1<br>1.0<br>3.0
ummumumuu	0<br>0.2<br>0.2	0<br>0.2<br>0.2	1<br>0.2<br>2.2	0<br>0.2<br>0.2	1<br>1.0<br>3.0
ummumuumm	0<br>0.2<br>0.2	0<br>0.2<br>0.2	0<br>0.2<br>0.2	1<br>0.2<br>2.2	1<br>1.0<br>3.0
ummumuumu	0<br>0.2<br>0.2	1<br>0.2<br>2.2	0<br>0.2<br>0.2	0<br>0.2<br>0.2	1<br>1.0<br>3.0

ummuummuu	0<br>0.2<br>0.2	0<br>0.2<br>0.2	0<br>0.2<br>0.2	1<br>0.2<br>2.2	1<br>1.0<br>3.0
ummuumuuu	0<br>0.2<br>0.2	0<br>0.2<br>0.2	0<br>0.2<br>0.2	1<br>0.2<br>2.2	1<br>1.0<br>3.0
ummuuumum	0<br>0.2<br>0.2	1<br>0.2<br>2.2	0<br>0.2<br>0.2	0<br>0.2<br>0.2	1<br>1.0<br>3.0
ummuuumuu	1<br>0.2<br>2.2	0<br>0.2<br>0.2	0<br>0.2<br>0.2	0<br>0.2<br>0.2	1<br>1.0<br>3.0
ummuuuuuu	0<br>0.2<br>0.2	0<br>0.2<br>0.2	1<br>0.2<br>2.2	0<br>0.2<br>0.2	1<br>1.0<br>3.0
umummmmuu	0<br>0.2<br>0.2	0<br>0.2<br>0.2	0<br>0.2<br>0.2	1<br>0.2<br>2.2	1<br>1.0<br>3.0
umumummuu	0<br>0.2<br>0.2	0<br>0.2<br>0.2	1<br>0.2<br>2.2	0<br>0.2<br>0.2	1<br>1.0<br>3.0
umumuumum	1<br>0.2	0<br>0.2	0<br>0.2	0<br>0.2	1<br>1.0

	2.2	0.2	0.2	0.2	3.0
umumuuumm	1	0	0	0	1
	0.2	0.2	0.2	0.2	1.0
	2.2	0.2	0.2	0.2	3.0
umumuuumu	0	0	0	1	1
	0.2	0.2	0.2	0.2	1.0
	0.2	0.2	0.2	2.2	3.0
umuumumuu	0	0	0	1	1
	0.2	0.2	0.2	0.2	1.0
	0.2	0.2	0.2	2.2	3.0
umuumuumu	1	0	0	0	1
	0.2	0.2	0.2	0.2	1.0
	2.2	0.2	0.2	0.2	3.0
umuuummmm	0	0	1	0	1
	0.2	0.2	0.2	0.2	1.0
	0.2	0.2	2.2	0.2	3.0
umuuumumu	0	1	0	0	1
	0.2	0.2	0.2	0.2	1.0
	0.2	2.2	0.2	0.2	3.0
umuuuuumu	0	0	0	1	1
	0.2	0.2	0.2	0.2	1.0
	0.2	0.2	0.2	2.2	3.0
umuuuuuuu	0	1	0	0	1

	0.2<br>0.2	0.2<br>2.2	0.2<br>0.2	0.2<br>0.2	1.0<br>3.0
uummmuumm	0<br>0.2<br>0.2	0<br>0.2<br>0.2	0<br>0.2<br>0.2	1<br>0.2<br>2.2	1<br>1.0<br>3.0
uummmuuuu	0<br>0.2<br>0.2	1<br>0.2<br>2.2	0<br>0.2<br>0.2	0<br>0.2<br>0.2	1<br>1.0<br>3.0
uummuuumm	0<br>0.2<br>0.2	0<br>0.2<br>0.2	0<br>0.2<br>0.2	1<br>0.2<br>2.2	1<br>1.0<br>3.0
uumummumu	0<br>0.2<br>0.2	1<br>0.2<br>2.2	0<br>0.2<br>0.2	0<br>0.2<br>0.2	1<br>1.0<br>3.0
uumumummu	0<br>0.2<br>0.2	0<br>0.2<br>0.2	1<br>0.2<br>2.2	0<br>0.2<br>0.2	1<br>1.0<br>3.0
uumumumuu	0<br>0.2<br>0.2	0<br>0.2<br>0.2	0<br>0.2<br>0.2	1<br>0.2<br>2.2	1<br>1.0<br>3.0
uumuummum	0<br>0.2<br>0.2	0<br>0.2<br>0.2	1<br>0.2<br>2.2	0<br>0.2<br>0.2	1<br>1.0<br>3.0

uumuumuum	1	0	0	0	1
	0.2	0.2	0.2	0.2	1.0
	2.2	0.2	0.2	0.2	3.0
uumuuummm	0	1	0	0	1
	0.2	0.2	0.2	0.2	1.0
	0.2	2.2	0.2	0.2	3.0
uumuuummu	1	0	0	0	1
	0.2	0.2	0.2	0.2	1.0
	2.2	0.2	0.2	0.2	3.0
uumuuumum	1	0	0	0	1
	0.2	0.2	0.2	0.2	1.0
	2.2	0.2	0.2	0.2	3.0
uuummmmuu	0	1	0	0	1
	0.2	0.2	0.2	0.2	1.0
	0.2	2.2	0.2	0.2	3.0
uuummumum	1	0	0	0	1
	0.2	0.2	0.2	0.2	1.0
	2.2	0.2	0.2	0.2	3.0
uuumummmm	0	0	1	0	1
	0.2	0.2	0.2	0.2	1.0
	0.2	0.2	2.2	0.2	3.0
uuumummum	1	0	0	0	1
	0.2	0.2	0.2	0.2	1.0
	2.2	0.2	0.2	0.2	3.0

uuuummmmu	0<br>0.2<br>0.2	0<br>0.2<br>0.2	0<br>0.2<br>0.2	1<br>0.2<br>2.2	1<br>1.0<br>3.0
uuuummuum	0<br>0.2<br>0.2	0<br>0.2<br>0.2	0<br>0.2<br>0.2	1<br>0.2<br>2.2	1<br>1.0<br>3.0
uuuummuuu	1<br>0.2<br>2.2	0<br>0.2<br>0.2	0<br>0.2<br>0.2	0<br>0.2<br>0.2	1<br>1.0<br>3.0
uuuumummm	0<br>0.2<br>0.2	1<br>0.2<br>2.2	0<br>0.2<br>0.2	0<br>0.2<br>0.2	1<br>1.0<br>3.0
uuuumummu	0<br>0.2<br>0.2	0<br>0.2<br>0.2	0<br>0.2<br>0.2	1<br>0.2<br>2.2	1<br>1.0<br>3.0
uuuumumuu	0<br>0.2<br>0.2	0<br>0.2<br>0.2	1<br>0.2<br>2.2	0<br>0.2<br>0.2	1<br>1.0<br>3.0
uuuuummmm	1<br>0.2<br>2.2	0<br>0.2<br>0.2	0<br>0.2<br>0.2	0<br>0.2<br>0.2	1<br>1.0<br>3.0
uuuuumumu	0<br>0.2	1<br>0.2	0<br>0.2	0<br>0.2	1<br>1.0

	0.2	2.2	0.2	0.2	3.0
uuuuuummm	0	0	1	0	1
	0.2	0.2	0.2	0.2	1.0
	0.2	0.2	2.2	0.2	3.0
uuuuuumum	0	0	0	1	1
	0.2	0.2	0.2	0.2	1.0
	0.2	0.2	0.2	2.2	3.0
Total	30	30	30	30	120
	30.0	30.0	30.0	30.0	120.0
	76.3	74.3	81.0	79.0	310.7

Pearson chi2(**315**) = **310.6667**   Pr = **0.558**
