# Supplementary material for: Promoter Methylation Pattern Controls Corticotropin Releasing Hormone Gene Activity in Human Trophoblasts
Source: PLoS One. 2017 Feb 2;12(2):e0170671. doi: 10.1371/journal.pone.0170671 (PMC5289476; doi:10.1371/journal.pone.0170671)
Supplement: S2 Table — (PDF) [file pone.0170671.s005.pdf]

Supplementary Table 2

**Methylation levels in CRH promoter copies associated with transcription factors and modified histones**			
**Treatment**	ChIP-selection	% CpG methylation (meCpG/total CpG)	Significance
Vehicle	No-ChIP	51.85 % (140/270)	
8-Br-cAMP	No-ChIP	47.41 % (128/270)	
Vehicle	TBP	60.92 % (159/261)	**P=0.036***
8-Br-cAMP	TBP	60.93 % (170/279)	**P=0.002#**
8-Br-cAMP	Pol-II	92.35 % (507/549)	**P<0.001#**
8-Br-cAMP	pCREB	55.56 % (150/270)	P=0.070#
8-Br-cAMP	Acetyl-H3	81.48 % (220/270)	**P<0.001#**
8-Br-cAMP	Acetyl-H4	35.63 % (93/261)	**P=0.006#**
8-Br-cAMP	H3K4me3	25.19 % (68/270)	**P<0.001#**
8-Br-cAMP	H3K9me3	33.73 % (85/252)	**P=0.002#**
8-Br-cAMP	H3K27me3	40.74 % (110/270)	P=0.141#

Primary cytotrophoblasts cultures were treated with 250 μM 8-Br-cAMP or vehicle for 72h. Transcription factor- and modified histone-associated DNA was isolated by chromatin immunoprecipitation (ChIP), and CpG methylation in the CRH proximal promoter was determined by clonal bisulfite sequencing. Significance was tested by Fisher's exact test against non-imunoprecipitated (no-ChIP control) DNA from vehicle-treated cells (*), or against no-ChIP DNA from 8-Br-cAMP-treated cells (#). Values below our significance threshold (0.05) are highlighted in bold. Total CpG site number is calculated as CpG/promoter (=9) x promoter copies sequenced (= 8-11 /placenta in 3 placentae or 6 placentae in the case of Pol-II).
