# Supplementary material for: Promoter Methylation Pattern Controls Corticotropin Releasing Hormone Gene Activity in Human Trophoblasts
Source: PLoS One. 2017 Feb 2;12(2):e0170671. doi: 10.1371/journal.pone.0170671 (PMC5289476; doi:10.1371/journal.pone.0170671)
Supplement: S3 Table — (PDF) [file pone.0170671.s006.pdf]

Supplementary Table 2

Primer sequences

PCR Primers for Bisulfite-Converted DNA[1]

First PCR	Forward:	TTTGGGAAATTTTATTTAAGAATTTTT
	Reverse:	CTAAATTTCTCCACTCCAAAACCTA
Second (nested) PCR	Forward:	GTTAATGGATAAGTTATAAGAAGTTTTT
	Reverse:	TCCACTCCAAAACCTAAAATAAAAT

PCR Primers for ChIP-Scanning[1]
(GenBank: NG_016127.1)

CRH1	Forward:	GAAATGGATATGGCAACTTACAAAGA
	Reverse:	AATATGTACCTCCTTTGCTGGACAGT
CRH2	Forward:	CACACTTGGGAAATCTCATTCAAG
	Reverse:	GGTGACGTCAACGAGCCCTA
CRH3	Forward:	TTCCATTTTAG GGCTCGTTGAC
	Reverse:	GAATCTCACATCCAATTATATCAACAGAT
CRH4	Forward:	GGCAGGGCCCTATGATTTATG
	Reverse:	CGCTCTCTTGACAGCTCGATT
CRH5	Forward:	GGCAAATGCTGCGTGGTT
	Reverse:	CAGAGCCTGGAGTGGGATTTT

qPCR Primers for cDNA Amplification[2]
(GenBank NM_000756.3)

Forward:	TCCCATCTCCCTGGATCTCAC
Reverse:	GTGAGCTTGCTGTGCTAACTGCT

Sequencing Primers[3]

Forward:	TATTTAGGTGACACTATAG
Reverse:	TAATACGACTCACTATAGGG

[1] Primers were used at 400 nM, each
[2] Forward and reverse primers were used at 600 nM and 300 nM, respectively
[3] Used as per the manufacturer's instructions (Promega)
