# Supplementary material for: Promoter Methylation Pattern Controls Corticotropin Releasing Hormone Gene Activity in Human Trophoblasts
Source: PLoS One. 2017 Feb 2;12(2):e0170671. doi: 10.1371/journal.pone.0170671 (PMC5289476; doi:10.1371/journal.pone.0170671)
Supplement: S1 File — (PDF) [file pone.0170671.s007.pdf]

Supplementary Methods

Cytotrophoblast Primary Cultures

Cytotrophoblasts were isolated [1] from placentas delivered spontaneously at term after uncomplicated singleton pregnancies. Cotyledons (approximately 35g) were dissected of blood vessels and decidua and rinsed thoroughly with phosphate-buffered saline (PBS; 1.5mM KH_2PO_4, 2.7mM KCl, 8.9mM Na_2HPO_4, 136.9mM NaCl) containing gentamicin (50 mg/L). Minced tissues were digested in 150 ml of 1mg/ml Trypsin 2X (Worthington/'ScimaR', Templestowe, Victoria, Australia), 0.13mg/ml DNase I (Thermo Fisher Scientific, Scoresby, Victoria, Australia) in Ca-Mg free Hank's balanced salt solution supplemented with 20mM HEPES pH=7.4 and 50 mg/L gentamicin (HEPES-buffered Hank's). The tissues were digested for 25 min at 37°C with gentle agitation, allowed to settle for 1 min and the supernatant was carefully removed. Fresh enzyme solution (100 ml) was added to the tissue mince for a subsequent round of digestion. Phase contrast microscopy of the supernatant showed no release of trophoblast cells after the first and second digestions; therefore these supernatants were discarded. Supernatants containing trophoblast cells were collected from the third to the eighth digestions (75 ml enzyme solution, each) and sedimented at 800g for 5 min. Cell pellets were suspended in 5 ml of Trypsin inhibitor (Type-IS, Sigma-Aldrich, Castle Hill, NSW, Australia) solution (1 mg/ml in HEPES-buffered Hank's). The suspended cell pellets were pooled and passed through a 90μm pore size nylon screen to remove tissue debris. The filtered cells were centrifuged at 800 g for 7 min, suspended in 0.5ml fetal bovine serum (FBS) and incubated at room temperature for 10 min. Culture medium (DMEM high glucose supplemented with 3.7g/L sodium

bicarbonate, 0.11g/L sodium pyruvate, 25mM HEPES, 20% FBS and 50mg/L gentamicin, from Sigma-Aldrich SAFC) was added to make up the volume to 10 ml. Cytotrophoblasts were separated by density gradient centrifugation, as follows. Percoll stock (Sigma-Aldrich) was mixed with 0.11 volume of 10 × HEPES-buffered Hank's to make 90% Percoll, which was diluted to 5-70% with HEPES-buffered Hank's solution in 5 % steps and layered in 50 ml tubes as described [1] using a peristaltic pump. Percoll gradients with the cell suspension loaded on top were centrifuged at 1200 g at room temperature for 20 min. Trophoblast were collected from the 35~50% layers, resuspended in culture medium and sedimented at 800 g for 10 min. The final cell pellet was suspended in culture medium and counted using trypan blue staining and a haemocytometer. Cell viability was 85–90%. A portion of the cell suspension was processed as fresh cells, while the rest was plated at a density of 3×10^6 /well in six-well plates or 2.5×10^7 in $10cm^2$ culture dishes and cultured at 37°C in a 5% CO_2 atmosphere. Medium was removed after the initial 16h of culture (0h treatment), and fresh medium with vehicle or 250 micromoles/L 8-Br-cAMP was added for up to an additional 72h. No toxic effect of 8-Br-cAMP has been detected under these conditions [2]. As demonstrated by Kliman et al., [1] the cytotrophoblasts aggregated and subsequently syncytialised during this culture period.

Clonal bisulfite sequencing of the CRH Proximal Promoter

Our previously described method [3] was used, as follows. Genomic DNA was extracted from cells using the QIAamp DNA Mini Kit (Qiagen, Chadstone Centre, VIC, Australia), converted by the methylSEQr Bisulfite Conversion Kit (Applied Biosystems/ Thermo Fisher Scientific, Scoresby, Victoria, Australia) and amplified by two sequential PCR reactions using two pairs of nested primers

(Supplementary Table 3). The promoter fragment was separated by agarose gel electrophoresis, purified using the Wizard SV Gel and PCR Clean-Up System (Promega, Auburn, VIC, Australia) and ligated into the pGEM-T Easy Vector (Promega). JM109 Competent cells (Promega) were transformed with the ligated plasmids, and at least 20 white colonies from each plasmid preparation (generated by a particular trophoblast DNA sample) were processed for plasmid isolation using the GenElute plasmid Miniprep Kit (Sigma). After verifying the presence of inserts by restriction enzyme digestion, plasmid DNA was sequenced by the Australian Genome Research Facility (AGRF, Brisbane) using the dideoxy method. Every plasmid was sequenced from both the sense and anti-sense directions. Primer sequences (Supplementary Table 3) were provided by the plasmid supplier (Promega). Sequence information was processed using the BiQ Analyzer software [4] to determine protected cytosines in the CRH proximal promoter.

Chromatin Immunoprecipitation

Cells maintained in culture dishes were rinsed with PBS and crosslinked with 1% formaldehyde in PBS at room temperature for 10 min. The reaction was stopped by 125 mM glycine, and the cells were lysed in 500 µl of Lysis Buffer (1% SDS, 10 mM EDTA, 50 mM TrisHCl, pH 8.1 with freshly added 0.1mM PMSF, 0.1mM Na_3VO_4, 1× cOmlplete protease inhibitor cocktail and 1× PhosSTOP phophatase inhibitor cocktail from Roche/Sigma-Aldrich). Cell lysates were collected in Eppendorf DNA LoBind tubes and sheared using the Misonix XL-2000 Sonicator (Qsonica, Newton, CT. USA). The sonication protocol, optimised in preliminary experiments, was 10 cycles of 10s

sonication and 50s rests with power set to 10 generating DNA fragments of 300 - 600 bp.

The supernatants of the sonicated cell lysates were distributed into 100 μl aliquots with a 50 μl portion of each preserved to serve as non-immunoprecipitated (NIP) input. The aliquots were diluted five-fold with Dilution Buffer (1% v/v Triton X-100, 2 mM EDTA, 150 mM NaCl, 20 mM Tris-HCl, pH 8.1, supplemented with 0.1% NP-40 v/v, protease inhibitor cocktail, phophatase inhibitor cocktail and 2μg salmon sperm DNA), and Protein A-agarose beads were added (20 μl, Santa Cruz Biotech/Thermo Fisher Scientific, Scoresby, VIC, Australia). The mixtures were incubated at 4°C for 2h as a preclearing step. Agarose beads were removed, and immunoprecipitation was performed by incubating the extracts overnight at 4°C with ChIP-grade antibodies or normal rabbit IgG antibody as non-specific control. The antibodies were used at concentrations optimised in preliminary experiments, as follows: from Santa Cruz Biotechnology: Pol-II (N-20) sc899, 5 μg; TFIID (TBP, SI-1) sc273, 3 μg; pCREB1 (Ser-133) sc7978R, 5 μg; normal rabbit IgG sc2027, 5 μg; from Merck Millipore (Bayswater, VIC, Australia): acetyl histone-3 06-599, 5 μg; acetyl histone-4 06-598, 5 μg; trimethyl histone-3 (Lys-4) 07-473, 5 μg; trimethyl histone-3 (Lys-27) ABE44, 5 μg; from Abcam (Melbourne, VIC, Australia): trimethyl histone-3 (Lys-9) ab8898, 5 μg. Samples were supplemented with 2 μg of salmon sperm DNA, and the immune complexes were captured with 40 μl of two-fold diluted Protein A-agarose beads pre-equilibrated overnight with a mixture of 400ul dilution buffer, 100ul lysis buffer, 50 μg BSA and 2ug salmon sperm DNA. After 2 h incubation at 4°C, the agarose beads were washed sequentially with TSE I buffer (0.1 % w/v SDS, 1% v/v Triton-X 100, 2 mM EDTA, 20mM Tris-HCl pH 8.1, 150 mM

NaCl), TSE II buffer (TSE I with NaCl concentration increased to 500 mM), Buffer 3 (0.25 M LiCl, 1 % v/v NP-40, 1% w/v deoxycholate, 1 mM EDTA, 10 mM Tris-HCl, pH 8), and 3 times with TE (10 mM Tris-HCl, pH8.0, 1 mM EDTA). The DNA-protein complexes were eluted from the Protein-A agarose beads sequentially three times with 150 µl, 100 µl and 100 µl Elution Buffer (Lysis Buffer supplemented with 0.1 M $NaHCO_3$) at 65°C for 10 min. The NIP sample was also adjusted to 0.1 M $NaHCO_3$, and crosslinks were reversed at 65°C overnight.

DNA eluted from Protein-A agarose was purified using the Wizard® SV Gel and PCR Clean-Up System (Promega), and CRH proximal promoter sequences were detected by real-time PCR. Five primer pairs were designed using Primer Express (Applied Biosystems/Thermo Fisher Scientific) to cover the CRE, TATA-I, TATA-II and the CDXARE regions of the promoter and an upstream control region (Table 1, Figure 2). The triplicate amplification reactions contained 5 µl of 5-fold diluted template DNA, 10 µl of 2× SYBR Green PCR master mix (Applied Biosystems) and 400 nM of forward and reverse primers, each, in a final volume of 20 µl. The amplification condition was 50°C for 2 min, 95°C for 10 min, 40 cycles of 95°C for 15 s and 60°C for 1 min. The NIP sample was used as control to measure abundance relative to input. No template control (NTC) was included to monitor assay background, and rabbit IgG control was used to detect any non-specific immunoprecipitation. A previously sonicated NIP sample was used as the calibrator to allow data comparison from multiple experiments. Relative abundance was calculated by the delta-delta Ct method [5].

1. Kliman HJ, Nestler JE, Sermasi E, Sanger JM, Strauss JF, 3rd. Purification, characterization, and in vitro differentiation of cytotrophoblasts from human term placentae. Endocrinology. 1986;118(4):1567-82.

2. Cheng YH, Nicholson RC, King B, Chan EC, Fitter JT, Smith R. Corticotropin-releasing hormone gene expression in primary placental cells is modulated by cyclic adenosine 3 ',5 '-monophosphate. J Clin Endocr Metab. 2000;85(3):1239-44.

3. Pan X, Bowman M, Scott RJ, Fitter J, Nicholson RC, Smith R, et al. Methylation of the Corticotropin Releasing Hormone Gene Promoter in BeWo Cells: Relationship to Gene Activity. International Journal of Endocrinology. 2015;2015:1-8.

4. Bock C, Reither S, Mikeska T, Paulsen M, Walter J, Lengauer T. BiQ Analyzer: visualization and quality control for DNA methylation data from bisulfite sequencing. Bioinformatics. 2005;21(21):4067-8.

5. Livak KJ, Schmittgen TD. Analysis of relative gene expression data using real-time quantitative PCR and the 2(-Delta Delta C(T)) Method. Methods. 2001;25(4):402-8.
